# Effects of Spice Treatment Methods on the Flavor Modification and Quality Characteristics of Surimi/Pea Protein Mixed Gels

**DOI:** 10.3390/foods15193407

**Published:** 2026-09-24

**Authors:** Tao Ye, Leman Bu, Yuchao Zhang, Mengying Ren, Hongtao Zhu, Zhina Chen, Lin Lin, Jianfeng Lu

**Affiliations:** 1 School of Bioengineering, Huainan Normal University, Huainan 232038, China; yetao430@163.com (T.Y.); 18155435940@163.com (L.B.); 2021030067@mail.hfut.edu.cn (M.R.); edi_zhu@163.com (H.Z.); chenzhina0625@163.com (Z.C.); 2 School of Food and Biological Engineering, Hefei University of Technology, Hefei 230009, China; linlin@hfut.edu.cn; 3 School of Chemical and Materials Engineering, Huainan Normal University, Huainan 232038, China; zhangyuchao215@163.com

**Keywords:** mixed gel, pea protein, flavor modification, ready-to-eat surimi product, spice

## Abstract

To develop a flavor-modification strategy for silver carp surimi products, pea protein was used to partially replace surimi protein at a substitution level of 30% (*w*/*w*) to prepare surimi/pea protein mixed gels (SPGs). Two spice treatment methods were compared: deodorized SPG prepared by adding spice water during gel preparation (DSPG-A) and deodorized SPG incubated in spice water during the second-stage heating (DSPG-I). The effects of these treatments on odor characteristics, volatile flavor composition, gel quality, and processing energy consumption were investigated. Sensory evaluation showed that DSPG-I was more effective in reducing fishy and beany odors and produced a braised flavor profile dominated by spicy aroma, suggesting its potential for flavor modification. Gas chromatography–ion mobility spectrometry (GC-IMS) further revealed that DSPG-I enriched characteristic spice-derived aroma compounds and showed increased intensities of roasted aroma-related compounds, including 2-furanmethanol acetate, and benzaldehyde. Quality analysis showed that no significant differences in gel strength or water-holding capacity were observed between the two spice-treated groups (DSPG-A and DSPG-I). Notably, DSPG-I significantly increased the surface a* and b* values of the mixed gels (*p* < 0.05), giving the products a characteristic color similar to that of braised products. A theoretical energy consumption estimation indicated that the comprehensive energy consumption for producing ready-to-eat surimi products using DSPG-I was 79.98 kgce/t, which was lower than that of the conventional process at 100.06 kgce/t, corresponding to theoretical energy-saving rate of approximately 20%. Overall, DSPG-I shows potential for application in the processing of ready-to-eat freshwater surimi products.

## 1. Introduction

Surimi is a concentrated aquatic myofibrillar protein prepared from fish through filleting, meat separation, washing to remove part of the water-soluble proteins and impurities, followed by dehydration and refining to improve protein purity [1]. After salt-assisted chopping, surimi forms a myofibrillar protein sol with heat-induced gelation properties, enabling further processing into shaped products such as fish balls, fish cakes, imitation crab meat, and fish rolls [2]. Combined with different storage and sterilization processes, surimi can also be developed into frozen prepared foods, ready-to-eat products, and other high-protein aquatic foods [3]. Owing to their desirable texture, nutritional value, and processing adaptability, surimi products have long been favored by consumers [4]. However, overfishing and the decline of marine fisheries have shifted the surimi industry’s focus from traditional marine fish toward the efficient utilization of freshwater resources [5].

Silver carp has become an important raw material for freshwater surimi processing because of its high yield, rapid growth, low cost, and abundant availability [6]. Nevertheless, silver carp surimi is generally characterized by a strong fishy odor, which severely limits its application in high-quality surimi products. The characteristic fishy odor compounds in freshwater surimi mainly include aldehydes, alcohols, and ketones. Among them, unsaturated aldehydes such as (Z)-4-heptenal, hexanal, nonanal, and (E, E)-2,4-decadienal make particularly important contributions to fishy odor [7]. These odor-active compounds are mainly derived from the autoxidative degradation of unsaturated fatty acids in fish muscle, including DHA, EPA, linoleic acid, and oleic acid, which generate volatile compounds such as aldehydes and alcohols [8].

In addition, microbial metabolism may produce sulfur-containing compounds, such as dimethyl trisulfide and methanethiol, further aggravating the off-odor of surimi products. Therefore, a fishy off-odor has become a major factor limiting quality improvement and consumer acceptance of freshwater surimi products [9].

In our previous work on the development of sustainable surimi products, partial replacement of surimi protein with pea protein was found to reduce the fishy odor of the resulting mixed gels to some extent. However, as the substitution level of plant protein increased, the characteristic fish flavor was also markedly diluted, leading to a weakened overall flavor profile. Sensory evaluation indicated that a 30% substitution level (*w*/*w*, protein/protein basis) was the critical threshold for maintaining an acceptable fish flavor. At this substitution level, the mixed gel retained a basic fish flavor, but fishy odor remained perceptible and had not been effectively eliminated [10].

The perceived intensity of fishy odor in surimi products is mainly determined by the release behavior of volatile and non-volatile off-odor compounds from the surimi gel matrix. This release process is jointly affected by the composition of odor compounds, food matrix components, and gel texture [11]. Currently, the use of food spices or their extracts to reduce the fishy odor of freshwater fish has become an important strategy for regulating the flavor quality of aquatic products. Spices have shown promising potential in both odor reduction and flavor reconstruction [12]. On the one hand, aroma compounds can alter the release rate of fishy odor compounds through physical masking, competitive adsorption, and binding interactions, thereby reducing consumers’ perception of fishy odor [13]. On the other hand, it has been reported that spices are rich in antioxidant components that could scavenge free radicals generated during lipid oxidation, inhibit the oxidative degradation of unsaturated fatty acids, and reduce the formation of certain fishy volatile compounds at the source, thereby improving the overall flavor quality of freshwater fish products [14]. A further study reported that major aroma compounds in spices, such as linalool, anethole, myrcene, and limonene, can bind to myofibrillar proteins through hydrophobic interactions and hydrogen bonding and competitively occupy the binding sites of fishy odor compounds, such as hexanal and 1-octen-3-ol, on proteins. Because these aroma compounds have lower binding energies, they can form more stable complexes with proteins during heating, increase the α-helix content, reduce surface hydrophobicity, and thereby promote the desorption and volatilization of fishy odor compounds, ultimately achieving deodorization [15]. Nevertheless, whether such aroma–protein interactions can be further enhanced by applying spice water as the external heating medium during gelation, rather than merely incorporating it into the formulation, remains unclear.

At present, studies on the application of spices in surimi products have mainly focused on the direct addition of spices during preparation or braising after gel formation. By contrast, the proposed immersion-heating strategy provides an alternative pathway for flavor incorporation, enabling simultaneous gelation and flavor modification in a single step. In this study, pea protein was used to partially replace surimi protein at a substitution level of 30% (*w*/*w*) to prepare surimi/pea protein mixed gels. The effects of spice water-assisted gelation on off-odor reduction, flavor reconstruction, and gel quality of the mixed gels were systematically investigated. In addition, the energy consumption of the newly developed process was estimated. This study aimed to develop and evaluate a spice water-assisted gelation process for freshwater surimi products, with emphasis on its deodorization efficiency, flavor improvement, gel quality, and energy-saving potential, thereby providing technical support for the high-value utilization of freshwater surimi resources.

## 2. Materials and Methods

### 2.1. Materials

Frozen silver carp surimi (AAA grade, protein content: 15.5%) was purchased from Honghu Jingli Aquatic Food Co., Ltd. (Honghu, China). After cold-chain transportation to the laboratory, the surimi was immediately cut into pieces, portioned, and stored at −18 °C until use. Pea protein isolate was obtained from Xi’an Meihe Biotechnology Co., Ltd. (Xi’an, China), with a protein content of 90.41% and a moisture content of 3.99%. Spices, including *Angelica dahurica* (root), *Lysimachia foenum-graecum* (whole plant), *Foeniculum vulgare* (fruit), *Murraya exotica* (leaf), *Syzygium aromaticum* (flower bud), *Kaempferia galanga* (rhizome), *Lanxangia tsaoko* (fruit), *Cinnamomum cassia* (bark), *Laurus nobilis* (leaf), *Nardostachys jatamansi* (rhizome), *and Cymbopogon citratus* (leaf) were purchased from Anhui Yonghui Supermarket Co., Ltd. (Hefei, China) in a single batch and were food-grade. Transglutaminase (TGase; enzyme activity: 1850 U/g) was purchased from Hefei Bomei Biotechnology Co., Ltd. (Hefei, China).

### 2.2. Preparation of Surimi/Pea Protein Mixed Gels

Surimi gels and surimi/pea protein mixed gels were prepared according to a previously reported method with slight modifications [16]. Briefly, frozen surimi was thawed at 4 °C for 16 h and pre-chopped using a blender (MJ-LZ225, Guangdong Midea Consumer Electric Manufacturing Co., Ltd., Foshan, China) at 2000 r/min for 15 s. Salt, pea protein, water or spice water, and TGase were then added, followed by further chopping at 2000 r/min for 20 s. The TGase dosage was 6 U/100 g surimi. In the mixed gel groups, pea protein accounted for 30% (*w*/*w*) of the total protein content.

The chopped surimi paste was vacuum-treated at −30 kPa for 3 min, stuffed into casings (polyamide-polyethylene, PA/PE), and subjected to two-stage heating to induce gelation. The samples were first heated in a thermostatic water bath (LC-HH, Shanghai Lichen Instrument Technology Co., Ltd., Shanghai, China) at 40 °C for 1 h and then heated at 90 °C for 30 min. The casings were retained for the SG, SPG, and DSPG-A groups throughout the two-stage heating. For the DSPG-I group, the casings were removed after the first-stage heating and before the second-stage heating. The preparation process of each gel sample is shown in Figure 1.

Four treatment groups were prepared. The control group (SG) was formulated with 150 g silver carp surimi and 3.75 g NaCl. The surimi/pea protein mixed gels (SPG) group was prepared by partially replacing surimi protein with pea protein at 30% (*w*/*w*) of the total protein content. The basic formulation of SPG was as follows: surimi, 150 g; salt, 5.25 g; water, 49.06 g; and pea protein powder, 11.02 g. For the deodorized SPG prepared by adding spice water during preparation (DSPG-A) group, spice water was used to replace water in the SPG formulation and was added during the chopping stage. For the deodorized SPG incubated in spice water during the second-stage heating (DSPG-I) group, the formulation was the same as that of SPG, but spice water was used as the heating medium during the second-stage heating process.

Based on preliminary experiments, the spice formulation and extraction procedure were determined as follows: *Angelica dahurica* (root, 5 g), *Lysimachia foenum-graecum* (whole plant, 5 g), *Foeniculum vulgare* (fruit, 2 g), *Murraya exotica* (leaf, 2 g), *Syzygium aromaticum* (flower bud, 1 g), *Kaempferia galanga* (rhizome, 2 g), *Lanxangia tsaoko* (fruit, 3 g), *Cinnamomum cassia* (bark, 3 g), *Laurus nobilis* (leaf, 2 g), *Nardostachys jatamansi* (rhizome, 2 g), and *Cymbopogon citratus* (leaf, 3 g) were added to 1000 mL of distilled water and extracted at 90 °C for 30 min. After extraction, the solution was cooled and filtered to obtain the spice water extract, which was used for preparing DSPG-A. For DSPG-I samples, the same spices were added directly to the water used for the second-stage heating (sample-to-liquid ratio 1:3, *w*/*v*), while the first-stage heating was performed in a water bath without added spices.

### 2.3. Quantitative Descriptive Analysis of Odor Characteristics of Mixed Gels

Quantitative descriptive analysis (QDA) was used to evaluate the odor characteristics and deodorization effects of the mixed gels [17]. The sensory panel consisted of 10 panelists, including five males and five females aged 22–24 years. All panelists completed 18 h of theoretical training (including basic theories of sensory evaluation, control of conditions for sensory evaluation, sensory evaluation methodologies, scaling techniques and experimental design, etc.) and 18 h of practical sensory training (including basic taste and odor recognition tests, difference tests, ranking tests, grading (scoring) tests, and descriptive analysis tests, etc.). Panelists were randomized across sessions and sample order was balanced using a Latin square design. Prior to evaluation, the samples, randomly sourced from the three independent batches prepared for each treatment were equilibrated at approximately 20 °C for 10 min. Approximately 60 g of each sample was placed on a white porcelain plate, coded with a random three-digit number, and presented to the panelists in randomized order. The evaluated attributes included spicy aroma, fishy odor, muddy odor, beany odor, meaty aroma, and fatty flavor. A 7-point scale was used, where 1 indicated extremely weak intensity and 7 indicated extremely strong intensity. Sensory evaluation was conducted in two independent sessions with the same trained panel. In each session, 10 panelists evaluated four gel samples, and the sensory data were expressed as the mean ± standard deviation. Significant differences among the four samples were determined by one-way analysis of variance (ANOVA), followed by Duncan’s multiple-range test (*p* < 0.05). Two-way ANOVA was used to examine the effects of sensory session and sample-by-session interaction on sensory scores, in order to evaluate the repeatability of the sensory panel. The sensory evaluation protocol was approved by the Institutional Academic Ethics Committee, with the approval number 2023-sgxy-030506.

### 2.4. Determination of Volatile Flavor Compounds in Mixed Gels by GC-IMS

Volatile flavor compounds in the mixed gels were analyzed according to a previously reported method with slight modifications [18]. The volatile compounds were analyzed using a FlavourSpec^®^ gas chromatography-ion mobility spectrometry (GC-IMS) system (G.A.S. Gesellschaft für Analytische Sensorsysteme mbH, Dortmund, Germany). Briefly, 2.5 g of sample was accurately weighed into a 20 mL headspace vial and incubated at 60 °C for 20 min. The injector temperature was set at 65 °C, and nitrogen was used as the carrier gas. A 500 μL aliquot of headspace gas was injected into an MXT-5 capillary column (15 m × 0.53 mm). The initial carrier gas flow rate was 2 mL/min and maintained for 2 min, then increased to 100 mL/min within 18 min. The drift gas flow rate was maintained at 150 mL/min. The column temperature was set at 60 °C, and the IMS ionization chamber temperature was set at 45 °C.

### 2.5. Determination of Gel Strength

The mixed gels were equilibrated at room temperature, blotted to remove surface moisture, and cut into cylindrical samples with a diameter of 2 cm and a height of 2.5 cm. Puncture testing was performed using a TA-XTplus Texture Analyzer (Stable Micro Systems Ltd., Surrey, UK) equipped with a P/0.25S probe. The test parameters were as follows: trigger force, 5 g; pre-test speed, 5 mm/s; test speed, 1 mm/s; and post-test speed, 1 mm/s. Breaking force and breaking distance were recorded. Gel strength was calculated using Equation (1):

(1) Gel strength (g·cm) = breaking force (g) × breaking distance (cm)

### 2.6. Determination of Water-Holding Capacity

Water-holding capacity was determined using a reported method [19]. The mixed gels were equilibrated at room temperature, blotted to remove surface moisture, and cut into slices approximately 5 mm thick. The initial weight was measured using an analytical balance (FA1104N, Shanghai Minqiao Precision Scientific Instrument Co., Ltd., Shanghai, China) with an accuracy of 0.001 g. Each sample was then placed between two layers of filter paper and compressed under a static load of 5 kg for 3 min. After compression, the sample was weighed again. Each sample was measured in triplicate. Water-holding capacity was calculated using Equation (2):

(2) Water-holding capacity (%) = [1 − (*m*_1_ − *m*_2_)/*m*_1_] × 100%

where *m*_1_ is the sample weight before compression (g), and *m*_2_ is the sample weight after compression (g).

### 2.7. Determination of Color and Whiteness

The mixed gels were equilibrated at room temperature, blotted to remove surface moisture, and cut into cylindrical samples with a diameter of 2 cm and a height of 2.5 cm. The surface and internal color parameters, including L*, a*, and b* values, were measured using a colorimeter (NR200XE, Shenzhen 3nh Technology Co., Ltd., Shenzhen, China) with a D_65_ illuminant, CIE 10° standard observer, and an 8 mm measuring aperture. Whiteness was calculated using Equation (3):

(3) $$\mathrm{Whiteness}\;=\;100\;-\;\sqrt{{\left(100\;-\;L^{*}\right)}^{2}\;+\;a^{*2}\;+\;b^{*2}}$$

### 2.8. Comparative Analysis of Energy Consumption in Ready-to-Eat Surimi Product Processing Under Different Deodorization Processes

To evaluate the effect of deodorization processes on the energy consumption of ready-to-eat surimi product production, a conventional process [20,21] was used as the control. The improved DSPG-I process developed in this study was used as the experimental process. A functional unit of 1 t of mixed gel product was adopted. Equipment selection and operating parameters were determined according to the process flow of each treatment, and the comprehensive energy consumption per unit product was calculated to compare the energy consumption differences between the two processes.

### 2.9. Statistical Analysis

All experiments were repeated at least three times using three independent batches, and results are expressed as mean ± standard deviation. Statistical analysis was performed using IBM SPSS Statistics 22.0 software (IBM Corp., Armonk, NY, USA). One-way analysis of variance (one-way ANOVA) was used to evaluate differences among treatment groups, followed by Duncan’s multiple range test. The significance level was set at *p* < 0.05. Figures were prepared using Origin 2020 software (OriginLab Corporation, Northampton, MA, USA). Volatile flavor compounds were identified by comparing their retention indices (RI) and drift times with those in the built-in GC-IMS library (NIST retention index database and G.A.S. drift time database). The Reporter and Gallery Plot plug-ins were used to construct differential comparison maps and fingerprint plots, respectively, to characterize differences in volatile flavor composition among treatment groups.

Principal component analysis (PCA) and nearest-neighbor fingerprint analysis were performed using the GC-IMS built-in software (version VOCal 0.4.03, G.A.S. Gesellschaft für Analytische Sensorsysteme mbH, Dortmund, Germany). The data matrix comprised the peak volumes of volatile compounds identified in each sample, with three replicates per treatment group. The nearest-neighbor algorithm calculates the Euclidean distance between every pair of samples and ranks them by similarity, enabling rapid comparison of volatile profiles among groups. Data were auto-scaled prior to PCA.

## 3. Results and Discussion

### 3.1. Quantitative Descriptive Analysis of the Odor Characteristics of Mixed Gels Treated with Different Spice Treatment Methods

QDA was used to evaluate the odor characteristics of the mixed gels. The deodorization effects of different spice treatment methods were systematically compared by quantifying the perceived intensities of key odor attributes, including fishy odor, muddy odor, fatty flavor, beany flavor, spicy aroma, and meaty aroma. As shown in Figure 2, the fishy odor and muddy odor scores of SG were 4.00 ± 0.67 and 3.50 ± 0.53, respectively, indicating that the untreated surimi gel had a distinct fishy odor accompanied by a certain degree of muddy odor. Compared with SG, SPG showed reduced fishy and muddy odor intensities. However, pea protein replacement also introduced a plant protein-derived beany odor, with the beany odor score increasing to 3.25 ± 0.48. These results suggest that partial replacement of surimi protein with pea protein can dilute fish-derived off-odors to some extent, but this strategy alone is insufficient to improve the overall flavor quality and may introduce additional undesirable odor attributes.

Both spice treatment methods, namely DSPG-A and DSPG-I, showed favorable deodorization and flavor-modulating effects. The fishy odor scores of DSPG-A and DSPG-I decreased to 1.75 ± 0.48 and 1.00 ± 0.00, respectively, which were markedly lower than those of SG and SPG. Meanwhile, both treatments effectively reduced the beany odor introduced by pea protein in SPG, with the beany odor score decreasing to a low level of 1.25 ± 0.48. These findings indicate that spice treatment can not only reduce fish-derived off-odors but also alleviate the beany odor caused by pea protein substitution, thereby achieving simultaneous control of fishy and beany odors.

Notably, the spice treatment method had a pronounced effect on aroma release intensity and deodorization efficiency. Compared with DSPG-A, in which spice water was directly added during preparation, DSPG-I, in which spice water was used as the gelation medium during the second-stage heating, exhibited a higher spicy aroma score of 5.75 ± 0.42, markedly higher than that of DSPG-A at 3.50 ± 0.53. At the same time, DSPG-I showed the lowest fishy odor score, at only 1.00 ± 0.00. This suggests that during heat-induced gel formation, spice water may act as both a heat-transfer and mass-transfer medium, possibly facilitating the migration, diffusion, and binding of volatile spice compounds within the gel matrix, although the specific mechanisms require further investigation. This process may enhance the masking and modulation of fishy and beany odors and form an odor profile dominated by spicy aromas. Overall, the spice treatment method significantly affected the deodorization performance of the mixed gels, with DSPG-I showing greater effectiveness in reducing fishy and beany odors and imparting a more pronounced braised flavor profile.

### 3.2. Effects of Spice Treatment Methods on the Volatile Flavor Composition of Mixed Gels

To further elucidate the effects of different spice treatment methods on the flavor characteristics of the mixed gels at the molecular level, gas chromatography–ion mobility spectrometry (GC-IMS) was used to analyze volatile organic compounds (VOCs) in the mixed gels from each treatment group. The VOC pseudo-color plots generated from GC-IMS data provided an intuitive overview of the overall differences in volatile compound distribution among the different treatments (Figure 3(A1)). The volatile profiles of SG and SPG were generally similar. However, after spice treatment, the VOC profiles of DSPG-A and DSPG-I changed markedly, indicating that spice treatment significantly reshaped the volatile flavor profile of the mixed gels. Meanwhile, the fingerprints of DSPG-A and DSPG-I were relatively similar, suggesting that both spice treatment methods introduced similar spice-derived volatile compounds. The difference map obtained by subtracting the SG profile as the reference further confirmed this trend (Figure 3(A2)).

To further analyze the similarities and differences in VOC composition among samples, PCA was performed based on the volatile compound data of each group (Figure 3(B1)). The results showed that samples from different treatment groups were distributed in distinct regions of the PCA score plot, indicating that their volatile profiles could effectively distinguish the different treatments [22]. The first two principal components (PC1 and PC2) accounted for 52% and 29% of the total variance, respectively, with a cumulative variance of 81%. In addition, k-nearest neighbor (k-NN) analysis based on Euclidean distance was used to evaluate the similarity of odor characteristics among different treatment groups (Figure 3(B2)). SG was the most distant from the other three groups, indicating the greatest difference in volatile flavor between pure surimi gel and the other treatments. SPG was located between SG and the spice-treated groups, suggesting that pea protein replacement had already altered the volatile composition. In contrast, DSPG-A and DSPG-I were located close to each other, indicating similar volatile characteristics. This result was consistent with the fingerprint patterns observed in Figure 3(A1). Overall, partial replacement of surimi protein with pea protein altered the volatile flavor composition of the mixed gels, while subsequent spice deodorization further and more substantially reconstructed the flavor profile.

To clarify the changes in specific volatile compounds, representative compounds in the GC-IMS fingerprints of each group were qualitatively and relatively quantitatively analyzed, and the results are shown in Table 1. In SG, the volatile compounds with high response intensities mainly included hexanal dimer (6122.66 ± 207.51), hexanal monomer (3792.09 ± 77.97), 1-penten-3-ol (2169.67 ± 26.51), and heptanal monomer (2065.43 ± 36.21). Among them, hexanal usually presents a grassy odor, 1-penten-3-ol is associated with mushroom-like and earthy notes, and heptanal has a grassy aroma. These compounds are commonly recognized as fishy or off-odor-related volatiles in freshwater surimi [23,24]. These results indicate that fishy odor-related volatile compounds dominated untreated SG, which was consistent with the higher fishy and muddy odor scores observed in the sensory evaluation.

Compared with SG, SPG showed increased response intensities of several aldehydes, including hexanal dimer, heptanal, heptanal dimer, pentanal, butanal, and nonanal. In addition, the response intensities of 2-heptanone and ethyl acetate also increased significantly (*p* < 0.05). These compounds are generally associated with grassy notes, lipid oxidation-related odors, and beany flavor characteristics [25,26]. Conversely, the response intensities of 1-penten-3-ol and 1-hydroxy-2-propanone decreased significantly in SPG, suggesting that pea protein replacement may reduce some muddy odor-related compounds while introducing or enhancing volatiles associated with plant protein and lipid oxidation. Therefore, the overall flavor profile of SPG was characterized by increased grassy notes and fatty flavor, together with a relative reduction in muddy odor.

After spice treatment, the response intensities of characteristic spice-derived flavor compounds, including 1,8-cineole monomer, 1,8-cineole dimer, 4-terpineol, and linalool, increased significantly in both DSPG-A and DSPG-I (*p* < 0.05). This may be attributed to the fact that key aroma compounds from the spices had effectively migrated into the mixed gel system and imparted distinct characteristic aromas to the products. Interestingly, some aldehydes generally considered to be key contributors to fishy odor did not decrease after spice treatment; instead, they showed an increasing trend. For example, the response intensity of hexanal dimer increased in the order SG (6122.66 ± 207.51), SPG (6885.83 ± 163.25), DSPG-A (7650.03 ± 198.11), and DSPG-I (9602.66 ± 236.32), showing a significant increasing trend of SG < SPG < DSPG-A < DSPG-I (*p* < 0.05). The heptanal monomer and heptanal dimer showed similar trends. These results suggest that pea protein replacement and spice treatment did not directly reduce the GC-IMS response intensities of fishy odor-related aldehydes such as hexanal and heptanal. This might be explained by the fact that the introduction of pea protein may have altered the protein composition, gel network structure, and protein–flavor interactions in the gel system, potentially influencing the release behavior of volatile aldehydes [27]. It has been reported that both matrix effects, arising from altered protein–odorant interactions and gel network modifications [28], and perceptual odor interactions between spice volatiles and fishy odorants may contribute to this apparent discrepancy [29]. Combined with the sensory results, it is speculated that the flavor improvement caused by spice treatment may not mainly depend on the direct reduction in fishy aldehydes, but rather on the aroma-imparting, masking, and flavor-reconstruction effects of characteristic spice volatiles [30].

Further comparison of the volatile profiles of DSPG-A and DSPG-I showed that DSPG-A had relatively higher response intensities of some characteristic spicy aroma compounds, such as eucalyptol and terpineol. This indicates that direct addition of spice water during chopping could impart a stronger spicy aroma to the product and provide a stronger masking effect against fishy odor. By contrast, although the response intensities of characteristic spicy aroma compounds in DSPG-I were slightly lower than those in DSPG-A, the response intensities of furans, including 2-pentylfuran and 2-furanmethanol acetate, were higher in DSPG-I, with the response intensity of 2-furanmethanol acetate reaching statistical significance (*p* < 0.05). These compounds are generally associated with heat-induced reactions, Maillard reactions, and lipid oxidation-derived flavor formation, and may contribute roasted, nutty, and cooked flavor notes to the product [31,32]. Therefore, compared with DSPG-A, DSPG-I not only introduced characteristic spicy aromas, but also exhibited a richer and more layered flavor profile. This could potentially be associated with the formation of cooked flavor and roasted aroma during thermal processing, though the underlying mechanisms remain to be elucidated.

In summary, the flavor of SG was mainly affected by volatile compounds associated with fishy and muddy odors. SPG introduced beany odor, grassy notes, and fatty flavor due to pea protein replacement. Spice treatment markedly improved the overall flavor of the mixed gels through aroma addition, odor masking, and flavor reconstruction. Among the two deodorization processes, DSPG-A mainly contributed a strong spicy aroma and a pronounced masking effect, whereas DSPG-I further imparted roasted and cooked flavor notes while maintaining the characteristic spicy aroma. Therefore, DSPG-I produced a more balanced aroma profile, which effectively modified the flavor of SPG, and was consistent with its superior sensory odor modification performance (Figure 2).

### 3.3. Effects of Spice Treatment Methods on Gel Strength of Mixed Gels

After clarifying the effects of spice treatment on the flavor characteristics of the mixed gels, the effects of different treatments on gel textural properties were further evaluated. As shown in Figure 4A–C, the gel strength of SG was 1056.04 ± 371.26 g·cm, while that of SPG was 1003.44 ± 136.12 g·cm. After the two spice deodorization treatments, the gel strengths of DSPG-A and DSPG-I were 848.32 ± 200.03 g·cm and 858.55 ± 210.46 g·cm, respectively. Although the gel strength of the spice-treated groups exhibited a slight decreasing trend relative to SG and SPG, no statistically significant differences were found among the spice-treated groups with respect to gel strength, breaking force, or breaking distance (*p* > 0.05). These results indicate that spice deodorization did not significantly weaken the mechanical properties of the mixed gels, suggesting that both DSPG-A and DSPG-I could improve flavor while maintaining the textural stability of the mixed gels.

### 3.4. Effects of Spice Treatment Methods on Water-Holding Capacity of Mixed Gels

Water-holding capacity is an important indicator for evaluating gel network stability and quality characteristics of surimi gels [19]. As shown in Figure 4D, the water-holding capacities of SG and SPG were 96.02% and 96.93%, respectively, with no significant difference between them (*p* > 0.05). This indicates that the mixed gel maintained a high water retention capacity at the 30% pea protein replacement level. After spice treatment, the water-holding capacity of DSPG-A decreased to 95.59%. Although it was not significantly different from that of SPG, it was significantly lower than that of SG (*p* < 0.05). The water-holding capacity of DSPG-I was 95.83%, with no significant difference from either SG or SPG (*p* > 0.05). These results suggest that spice treatment had only a limited effect on the water-holding capacity of the mixed gels. Among the two treatments, DSPG-I did not significantly reduce water-holding capacity and was more favorable for maintaining water retention in the gel system than DSPG-A.

### 3.5. Effects of Spice Treatment Methods on Color and Whiteness of Mixed Gels

The effects of spice treatment methods on the color of mixed gels are shown in Figure 5. In terms of surface whiteness, SG had a value of 70.90 ± 0.32, whereas SPG showed a significant decrease to 65.54 ± 0.35 (*p* < 0.05), indicating that pea protein replacement reduced the surface whiteness of the gel. After spice treatment, the surface whiteness of DSPG-A was 65.23 ± 0.41, with no significant difference from SPG (*p* > 0.05). In contrast, the surface whiteness of DSPG-I further decreased significantly to 48.55 ± 0.19 (*p* < 0.05), and the samples exhibited an obvious caramel-like or braised-product-like color (Figure 5B).

Further analysis of internal color showed that the different treatments had no significant effects on the internal whiteness or internal L*, a*, and b* values (*p* > 0.05) (Figure 5C), indicating that spice water immersion mainly changed the surface color of the gels without markedly affecting the internal color. For surface color parameters, DSPG-I significantly increased both surface a* and b* values (*p* < 0.05), with the most pronounced increase observed in b*, representing yellowness (Figure 5D). This suggests that using spice water as the gelation medium during the second-stage heating could impart a characteristic color similar to that of braised products. This may be attributed to the deposition of spice compounds on the gel surface. This color change matched the spice-dominated odor profile of DSPG-I and may also enhance the cooked appearance and product identity of ready-to-eat surimi products.

### 3.6. Energy Consumption Analysis of Ready-to-Eat Surimi Product Production Using the DSPG-I Process

The above results showed that DSPG-I effectively improved the fishy odor of surimi products, imparted a spice-dominated braised flavor and braised-product-like color, and had no significant negative effect on gel strength or water-holding capacity. Having identified DSPG-I as the superior method in the preliminary screening, we further benchmarked it against the conventional post-formation braising process [20,21] to assess its potential industrial application value. Notably, this process integrated gelation and spice-water immersion deodorization into the same heating stage, which may simplify processing. To preliminarily evaluate its energy-saving potential, this study compared the energy consumption of DSPG-I in comparison with the conventional ready-to-eat surimi product processing process [20,21], based on a theoretical annual production capacity of 1000 t of ready-to-eat silver carp surimi products.

Assumptions and boundary conditions. The following assumptions were adopted for this estimation: an annual production capacity of 1000 t, 300 working days per year, and 8 h of operation per day, yielding a designed daily output of 3334 kg and an hourly output of approximately 417 kg. Equipment selection for both the conventional process and DSPG-I was based on this theoretical production capacity (Table 2). The energy consumption parameters for each unit operation were derived from equipment specifications, assuming continuous operation at full capacity without accounting for start-up/shut-down losses, equipment idle time, or heat recovery systems. Steam consumption was estimated based on standard heat transfer calculations for batch heating processes, assuming no thermal energy recycling.

In the conventional process, the electricity consumption values for each unit operation were 12.0, 9.6, 10.8, 3.6, 10.35, 8.52, 28.8, and 16.2 kWh/t, corresponding to thawing, slicing, chopping, forming, gelation, seasoning, packaging, and sterilization, respectively. Steam consumption was mainly concentrated in the gelation, braising, and sterilization steps, with values of 225, 216, and 504 kg/t, respectively. Compared with the conventional process, DSPG-I had generally similar energy consumption parameters for the remaining unit operations. The major difference was that the independent braising step was eliminated and the spice-water immersion deodorization process was integrated into the second-stage heating and gelation process.

According to the General Principles for Calculation of Comprehensive Energy Consumption (GB/T 2589-2020 [33]), electricity and steam consumption were converted into standard coal equivalents, using conversion coefficients of 0.1229 kgce/kWh for electricity and 0.0929 kgce/kg for steam. The standard coal equivalent energy consumption of each unit operation is shown in Figure 6. Except for the braising step, the standard coal equivalent energy consumption values of the remaining steps in both processes were 1.48, 1.18, 1.33, 0.44, 22.17, 1.05, 3.54, and 48.81 kgce/t. In the conventional process, the braising step contributed 20.06 kgce/t, whereas this energy consumption was completely eliminated in DSPG-I by removing the independent braising step. The comprehensive calculation indicated that the total estimated energy consumption of the conventional process was 100.06 kgce/t, while that of DSPG-I was 79.98 kgce/t, corresponding to a theoretical energy-saving rate of approximately 20% under the assumptions of this study. It should be noted that this energy consumption analysis is based on theoretical calculations rather than industrial validation. Factors such as actual equipment utilization, heat recovery, and operational variations were not considered. Therefore, the reported energy-saving rate of approximately 20% should be considered as a theoretical upper limit under the idealized conditions assumed in this analysis, and its actual performance in industrial settings requires further confirmation through pilot- or full-scale validation.

## 4. Conclusions

In this study, surimi/pea protein mixed gels were prepared by partially replacing silver carp surimi protein with pea protein, and the effects of two spice treatment methods on deodorization efficiency, volatile flavor composition, gel quality, and processing energy consumption were compared. The results showed that DSPG-I effectively reduced fishy and beany odors in the mixed gels, likely through a flavor-masking mechanism, while imparting a spice-dominated braised flavor profile and a characteristic braised-product-like color, without causing significant negative effects on gel strength or water-holding capacity. GC-IMS analysis further revealed that DSPG-I not only enriched characteristic spice-derived aroma compounds but also showed increased intensities of roasted aroma-related compounds such as furans and benzaldehyde, resulting in a more complex flavor profile. Theoretical energy consumption estimation suggested that DSPG-I may offer energy-saving potential compared with the conventional process, but this remains to be validated in industrial settings. Overall, DSPG-I demonstrated advantages in odor modification, flavor quality, and gel properties. Given that the present study was conducted at the laboratory scale and the samples did not undergo the full ready-to-eat processing sequence, the practical applicability of this process to commercial ready-to-eat products requires further investigation. The mass transfer behavior of active spice components, their exposure dosage, and subsequent reaction mechanisms (e.g., antioxidant effects) will be investigated in future work to further elucidate the flavor improvement mechanism of the DSPG-I process.

## Figures and Tables

**Figure 1:**
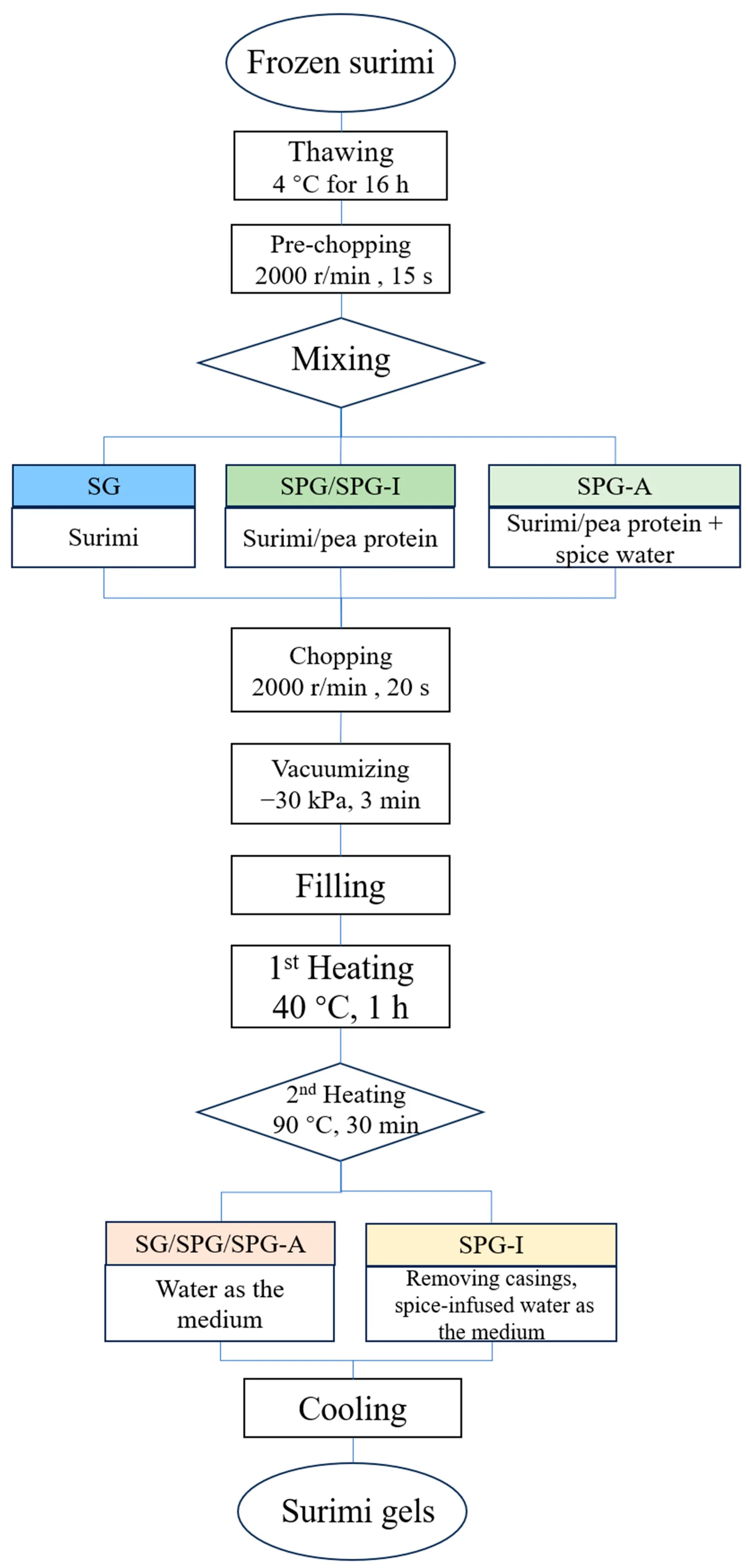
Flow diagram for the preparation of surimi/pea protein mixed gels.

**Figure 2:**
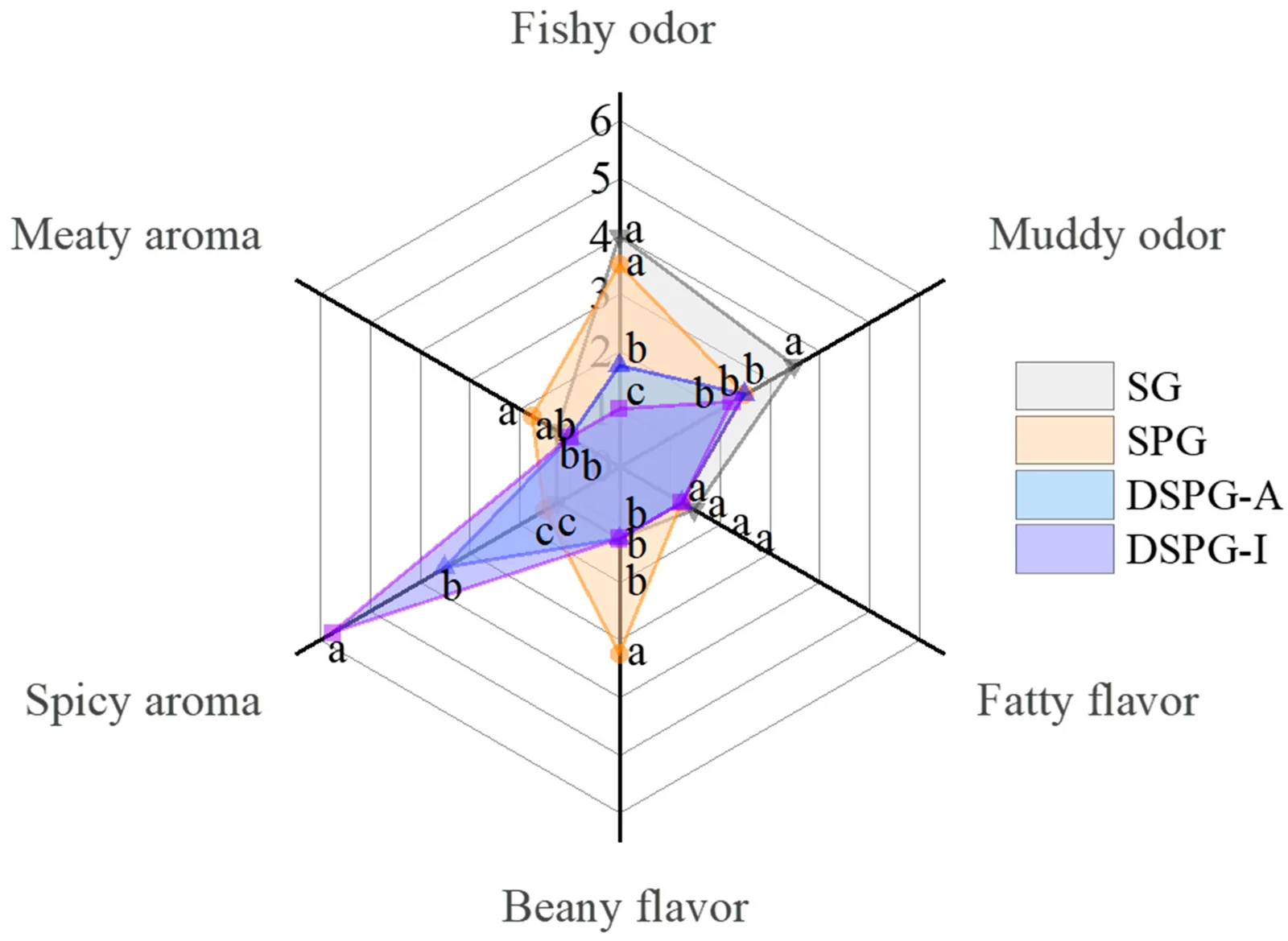
Effects of spice treatment methods on the odor characteristics of surimi/pea protein mixed gels evaluated by quantitative descriptive analysis. Different lowercase letters indicate significant differences (*p* < 0.05, *n* = 20).

**Figure 3:**
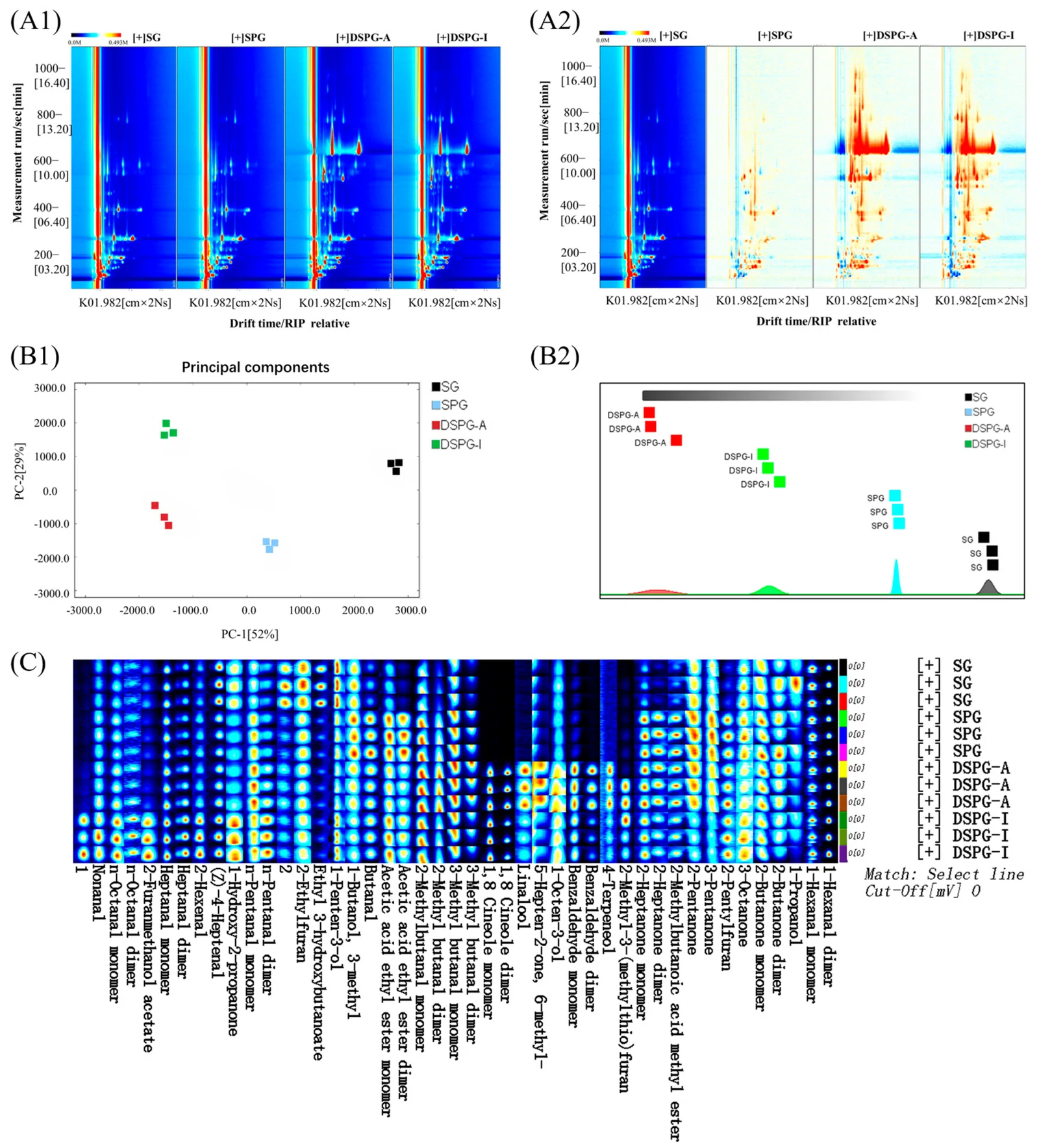
GC-IMS analysis of volatile organic compounds in surimi/pea protein mixed gels treated with different spice methods: pseudo-color plots (**A1**,**A2**), principal component analysis (**B1**), nearest-neighbor fingerprint analysis (**B2**), and fingerprint profiles (**C**).

**Figure 4:**
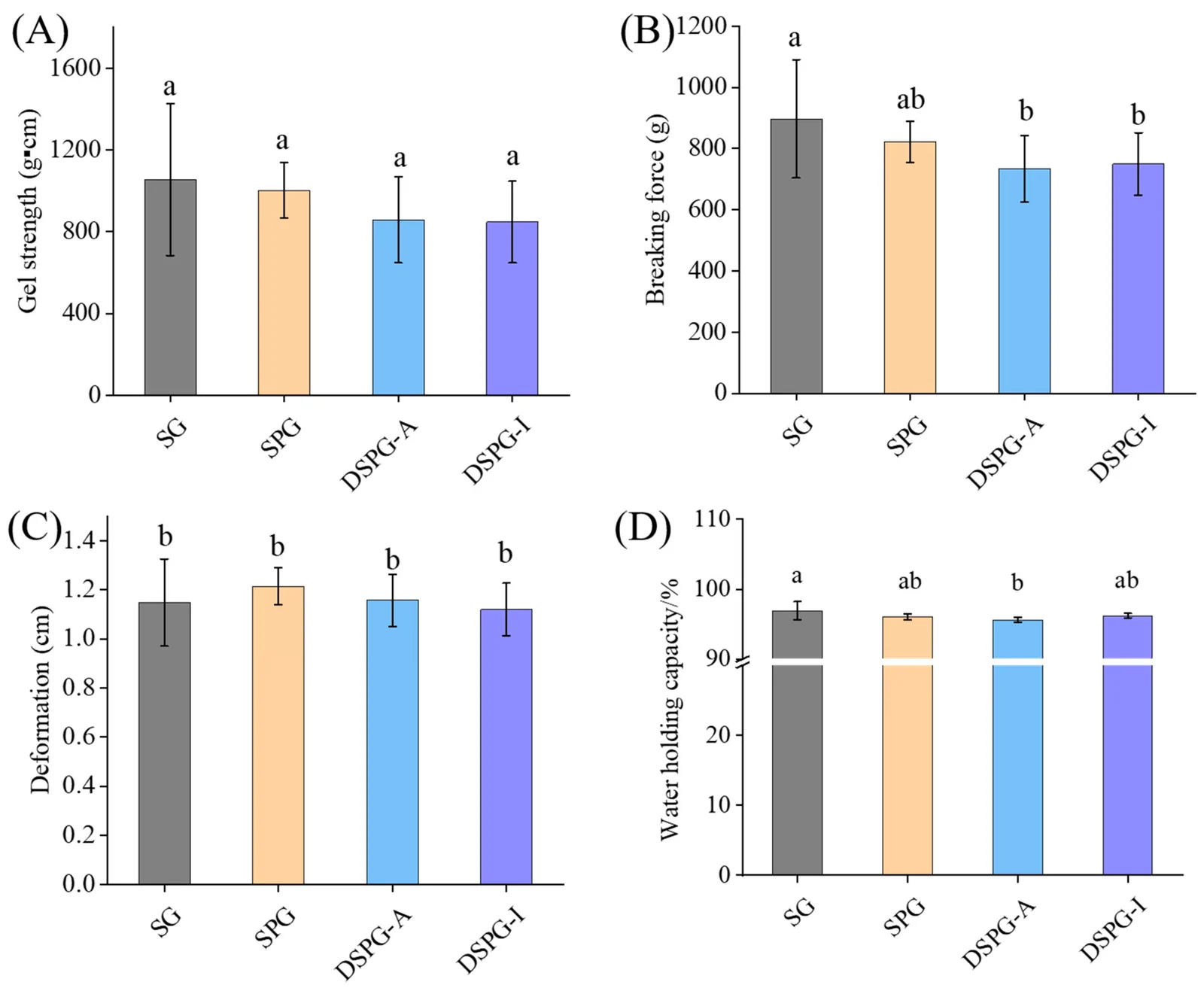
Effects of spice treatment methods on the textural properties and water-holding capacity of surimi/pea protein mixed gels: gel strength (**A**), breaking force (**B**), breaking distance (**C**), and water-holding capacity (**D**). Different lowercase letters above the bars indicate significant differences among treatments within the same panel (*p* < 0.05).

**Figure 5:**
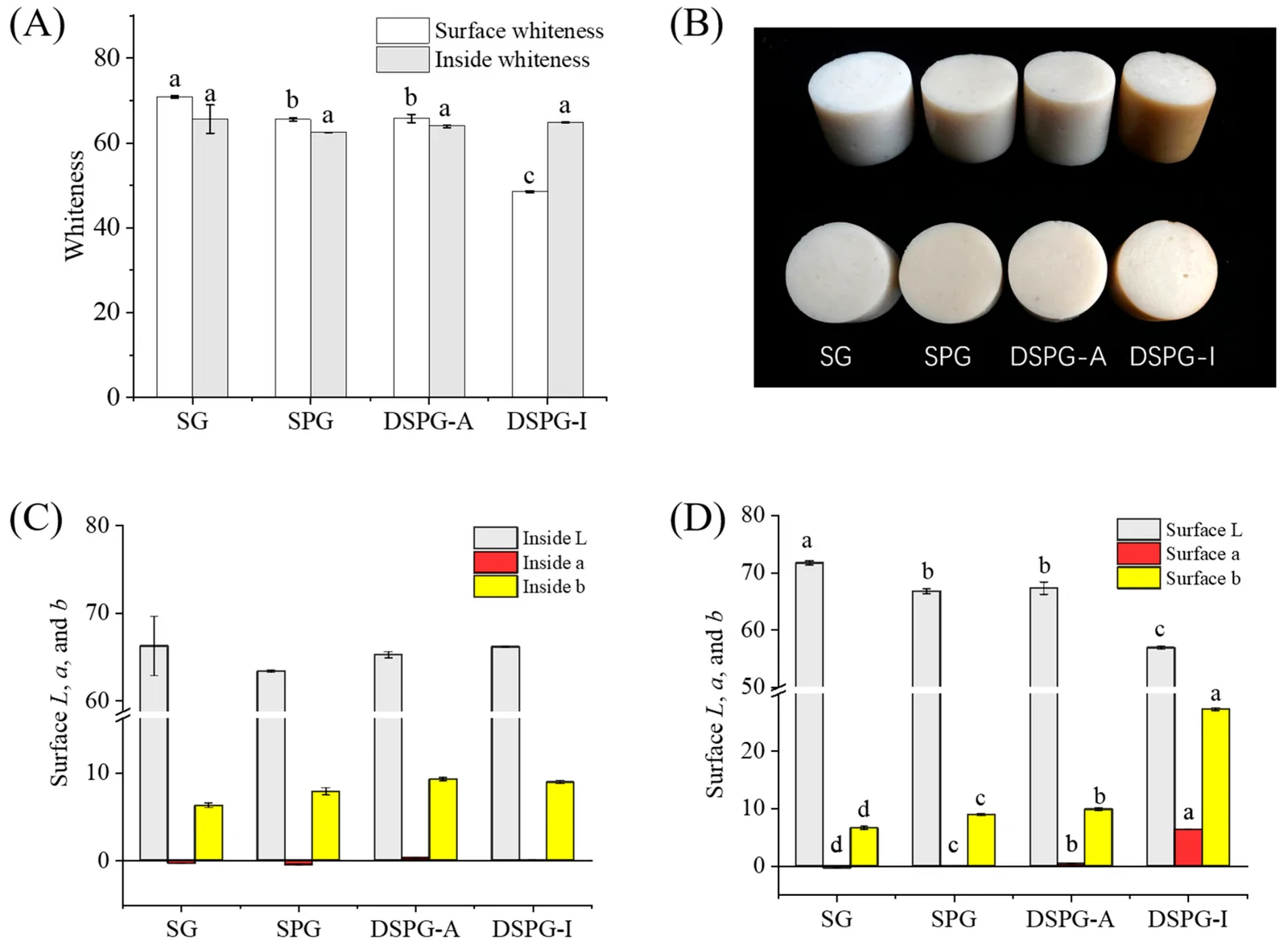
Effects of spice treatment methods on the whiteness, appearance, and color characteristics of surimi/pea protein mixed gels: whiteness (**A**), appearance (**B**), and color parameters (**C**,**D**). Different lowercase letters (a–c) above the bars indicate significant differences among treatments within the same panel (*p* < 0.05).

**Figure 6:**
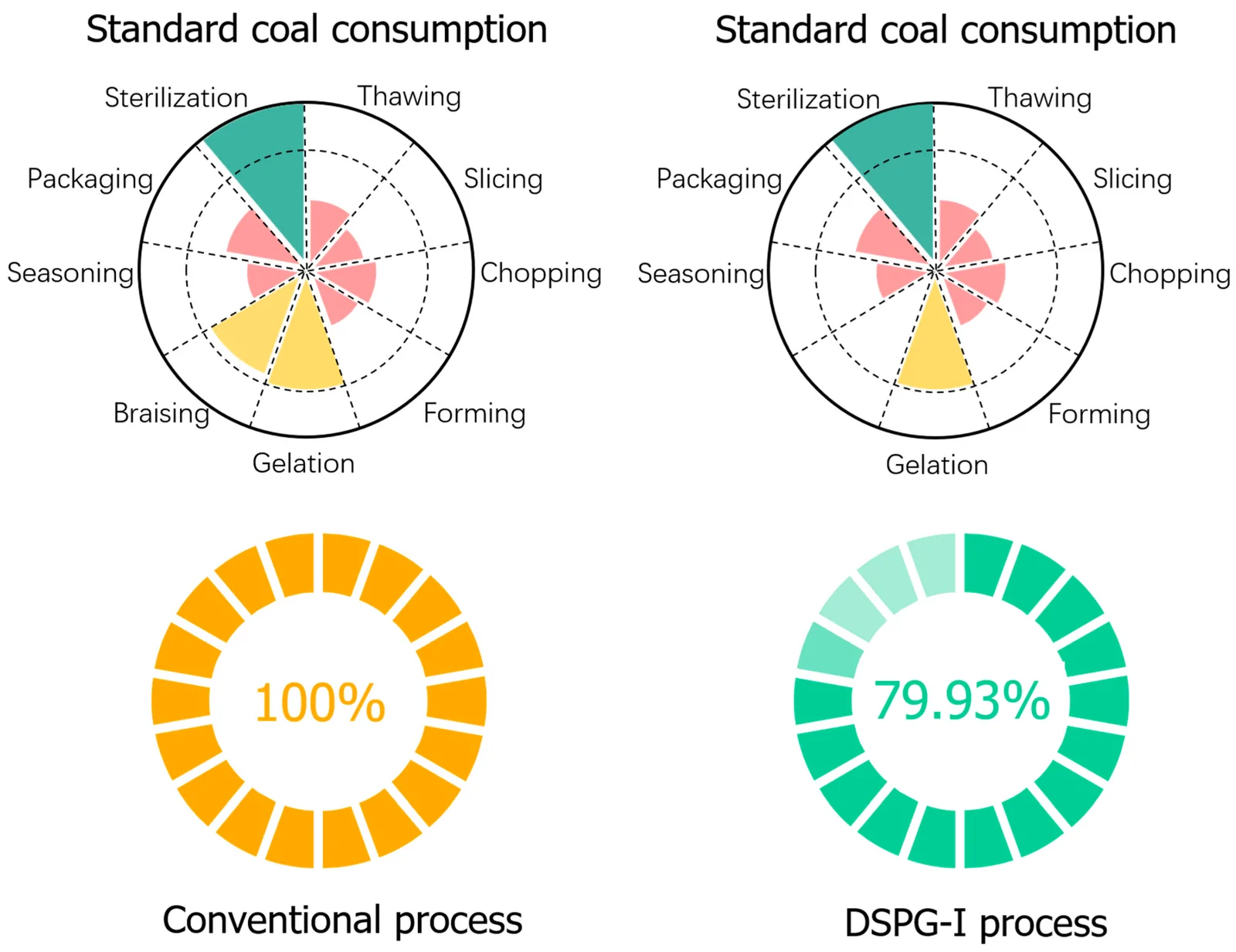
Comparison of comprehensive energy consumption per unit product between the conventional process and the DSPG-I process for ready-to-eat surimi products.

**Table 1:** Effects of spice treatment methods on the volatile flavor composition of surimi/pea protein mixed gels.

No.	Compound	RI	RT (s)	DT (a.u.)	SG	SPG	DSPG-A	DSPG-I
1	1,8-Cineole monomer	1043.3	652.44	1.293	1054.04 ± 85.55 ^c^	1220.65 ± 71.03 ^c^	20,385.29 ± 207.10 ^a^	17,915.93 ± 65.42 ^b^
2	1,8-Cineole dimer	1040.1	646.21	1.726	557.40 ± 80.09 ^c^	522.79 ± 31.88 ^c^	15,494.67 ± 255.24 ^a^	9252.13 ± 135.86 ^b^
3	4-Terpineol	1155.6	913.91	1.221	193.83 ± 34.78 ^c^	174.74 ± 9.42 ^c^	425.74 ± 34.53 ^a^	299.08 ± 23.84 ^b^
4	Linalool	1102.0	778.19	1.217	66.55 ± 2.96 ^c^	64.06 ± 1.94 ^c^	333.80 ± 18.42 ^a^	202.30 ± 13.51 ^b^
5	Nonanal	1099.0	771.20	1.472	305.09 ± 21.83 ^c^	396.18 ± 27.54 ^b^	429.68 ± 39.64 ^b^	567.33 ± 49.11 ^a^
6	Octanal monomer	1007.0	585.07	1.405	685.17 ± 12.89 ^c^	716.41 ± 54.81 ^bc^	777.56 ± 20.40 ^b^	1093.70 ± 28.75 ^a^
7	Octanal dimer	1005.6	582.62	1.819	202.49 ± 12.34 ^c^	203.86 ± 7.98 ^c^	257.70 ± 17.92 ^b^	310.35 ± 18.15 ^a^
8	2-Furanmethanol acetate	989.6	552.59	1.413	500.74 ± 18.09 ^b^	378.57 ± 46.49 ^c^	303.49 ± 36.56 ^c^	1292.04 ± 91.93 ^a^
9	6-Methyl-5-hepten-2-one	991.5	556.35	1.173	183.88 ± 10.17 ^c^	190.59 ± 12.22 ^c^	545.73 ± 6.92 ^a^	289.68 ± 30.98 ^b^
10	Benzaldehyde monomer	975.1	523.56	1.154	670.63 ± 103.10 ^d^	941.07 ± 25.10 ^c^	4046.25 ± 101.90 ^a^	2830.67 ± 92.66 ^b^
11	Benzaldehyde dimer	974.1	521.69	1.466	188.71 ± 8.60 ^c^	194.84 ± 14.46 ^c^	2247.88 ± 96.27 ^a^	1027.04 ± 15.12 ^b^
12	(Z)-4-Heptenal	894.1	387.73	1.145	629.47 ± 18.32 ^a^	475.47 ± 30.81 ^b^	490.68 ± 14.88 ^b^	624.64 ± 15.06 ^a^
13	Heptanal monomer	895.4	389.6	1.332	2065.43 ± 36.21 ^d^	2401.08 ± 98.52 ^c^	2647.10 ± 61.50 ^b^	2993.69 ± 70.25 ^a^
14	Heptanal dimer	895.4	389.6	1.695	759.62 ± 13.23 ^c^	855.05 ± 17.69 ^c^	1038.82 ± 84.45 ^b^	1450.82 ± 55.70 ^a^
15	2-Heptanone monomer	882.9	372.74	1.260	85.98 ± 6.14 ^c^	296.85 ± 30.83 ^a^	294.71 ± 3.97 ^a^	228.67 ± 7.13 ^b^
16	2-Heptanone dimer	880.8	369.93	1.627	380.70 ± 7.51 ^c^	682.89 ± 17.78 ^a^	694.63 ± 29.62 ^a^	615.56 ± 7.56 ^b^
17	Hexanal monomer	793.1	272.51	1.263	3792.09 ± 77.97 ^c^	3963.55 ± 60.86 ^b^	3922.19 ± 30.26 ^b^	4159.44 ± 78.28 ^a^
18	Hexanal dimer	789.1	268.76	1.558	6122.66 ± 207.51 ^d^	6885.83 ± 163.25 ^c^	7650.03 ± 198.11 ^b^	9602.66 ± 236.32 ^a^
19	3-Methyl-1-butanol	738.8	218.17	1.245	370.34 ± 11.34 ^b^	442.64 ± 7.54 ^a^	382.75 ± 10.12 ^b^	345.95 ± 11.68 ^c^
20	Pentanal monomer	696.3	182.41	1.191	541.95 ± 17.88 ^c^	668.35 ± 10.47 ^b^	751.42 ± 22.98 ^a^	753.90 ± 10.55 ^a^
21	Pentanal dimer	693.9	180.56	1.422	378.20 ± 23.50 ^d^	467.89 ± 40.56 ^c^	605.49 ± 35.03 ^b^	856.97 ± 22.97 ^a^
22	Ethyl acetate monomer	596.5	141.78	1.097	519.16 ± 14.90 ^d^	1052.25 ± 12.56 ^a^	849.62 ± 14.75 ^c^	1028.01 ± 4.18 ^b^
23	Ethyl acetate dimer	594.3	141.02	1.336	114.38 ± 11.99 ^d^	779.51 ± 52.38 ^a^	521.40 ± 22.83 ^c^	607.43 ± 21.40 ^b^
24	2-Butanone dimer	568.9	132.74	1.245	259.61 ± 49.96 ^a^	305.59 ± 62.23 ^a^	306.35 ± 27.68 ^a^	249.76 ± 40.26 ^a^
25	2-Butanone monomer	571.1	133.45	1.063	598.05 ± 12.10 ^a^	562.25 ± 18.40 ^ab^	535.14 ± 30.79 ^b^	532.93 ± 41.07 ^b^
26	Butanal	579.5	136.13	1.289	265.21 ± 33.20 ^c^	650.62 ± 51.17 ^a^	563.82 ± 18.94 ^b^	539.34 ± 44.87 ^b^
27	1-Hydroxy-2-propanone	694.7	181.15	1.226	381.38 ± 5.53 ^b^	274.79 ± 7.00 ^c^	298.49 ± 23.54 ^c^	487.65 ± 5.98 ^a^
28	2-Ethylfuran	696.1	182.26	1.305	256.88 ± 6.76 ^a^	200.48 ± 7.19 ^bc^	192.19 ± 1.85 ^c^	205.20 ± 4.62 ^b^
29	1-Octen-3-ol	993.8	561.31	1.156	770.28 ± 87.33 ^c^	762.06 ± 21.27 ^c^	1236.61 ± 83.22 ^a^	1100.54 ± 31.76 ^b^
30	2-Pentylfuran	990.0	553.3	1.251	108.99 ± 7.85 ^b^	238.50 ± 15.26 ^a^	238.43 ± 22.16 ^a^	258.24 ± 4.12 ^a^
31	2-Methyl-3-(methylthio)furan	955.5	486.86	1.148	82.66 ± 27.41 ^b^	112.07 ± 28.36 ^b^	623.07 ± 431.39 ^a^	658.01 ± 237.03 ^a^
32	2-Hexenal	843.7	325.08	1.183	220.18 ± 4.97 ^c^	246.13 ± 8.43 ^bc^	258.17 ± 19.18 ^b^	390.12 ± 18.43 ^a^
33	Methyl 2-methylbutanoate	771.1	250.04	1.19	142.54 ± 5.79 ^c^	233.26 ± 8.02 ^a^	222.26 ± 10.15 ^a^	202.33 ± 8.69 ^b^
34	2-Methylbutanal monomer	659.5	164.74	1.175	813.76 ± 11.92 ^c^	1094.21 ± 8.93 ^b^	1181.53 ± 14.57 ^a^	1079.50 ± 9.26 ^b^
35	2-Methylbutanal dimer	655.8	163.28	1.397	222.64 ± 20.51 ^d^	494.89 ± 26.06 ^b^	638.10 ± 16.79 ^a^	435.06 ± 13.79 ^c^
36	3-Methylbutanal monomer	641.9	157.97	1.179	903.79 ± 39.70 ^c^	1176.73 ± 6.65 ^b^	1258.47 ± 27.37 ^a^	1172.51 ± 31.06 ^b^
37	3-Methylbutanal dimer	645.2	159.22	1.401	206.11 ± 29.48 ^c^	427.71 ± 21.50 ^b^	572.54 ± 35.28 ^a^	368.58 ± 42.78 ^b^
38	1-Propanol	557.1	129.06	1.116	220.52 ± 81.78 ^a^	173.31 ± 60.34 ^a^	145.67 ± 20.01 ^a^	145.59 ± 63.96 ^a^
39	1-Penten-3-ol	691.0	178.34	0.945	2169.67 ± 26.51 ^a^	1588.50 ± 17.40 ^b^	1478.93 ± 46.86 ^c^	2117.91 ± 40.57 ^a^
40	2-Pentanone	681.5	173.62	1.121	103.73 ± 9.09 ^b^	124.12 ± 1.05 ^a^	120.07 ± 4.02 ^a^	77.47 ± 0.12 ^c^
41	3-Pentanone	697.3	183.18	1.116	135.79 ± 10.48 ^c^	190.68 ± 11.35 ^a^	155.77 ± 2.56 ^b^	117.59 ± 7.95 ^d^
42	Ethyl 3-hydroxybutanoate	938.3	456.8	1.173	289.18 ± 22.76 ^a^	166.16 ± 7.51 ^b^	155.68 ± 7.43 ^b^	166.81 ± 8.21 ^b^
43	3-Octanone	988.8	550.82	1.298	195.85 ± 9.55 ^ab^	225.00 ± 23.55 ^a^	150.13 ± 8.70 ^c^	175.53 ± 15.67 ^bc^

Values are expressed as mean ± standard deviation (*n* = 3). Different lowercase letters within the same row indicate significant differences among SG, SPG, DSPG-A, and DSPG-I (*p* < 0.05). RI, retention index; RT, retention time; DT, drift time.

**Table 2:** Equipment configuration and estimated energy consumption for a ready-to-eat surimi product production line with an annual capacity of 1000 t.

Process	Parameters	Equipment/Capacity	Power/Steam Consumption and Operating Time	Energy Consumption Based on Daily Production Output
Thawing	Thawed in an air-conditioned room at 5–10 °C until the core temperature reached −3 to 0 °C	Air-conditioning system for a room capacity of 72 m^3^	5 kW × 8 h, 1 unit	Electricity: 40 kWh
Slicing	Slice thickness: 5–10 mm	Automatic frozen meat slicer, processing capacity ≥ 417 kg/h	4.0 kW × 8 h, 1 unit	Electricity: 32 kWh
Bowl chopping	Dry chopping for 6 min, salt chopping for 18 min, and mixing/chopping for 6 min	Vacuum bowl cutter, 280 kg/batch, 12 batches in total	3 kW/unit × 2 units × 0.5 h/batch × 12 batches	Electricity: 36 kWh
Forming	Diameter: 26 mm	Surimi forming machine, processing capacity ≥ 417 kg/h	0.75 kW/unit × 2 units × 8 h	Electricity: 12 kWh
Gelation	Water-bath heating at 40 °C for 1 h followed by heating at 90 °C for 30 min	Sectional water-bath heating equipment, 667 kg/batch, 5 batches in total	4.6 kW × 1.5 h/batch × 5 batches; Steam: 100 kg/h × 1.5 h/batch × 5 batches	Electricity: 34.5 kWh; Steam: 750 kg
Braising *	Braising in a jacketed kettle for 30 min	Steam-jacketed kettle, 280 kg/batch, 12 batches in total	Steam: 60 kg/batch × 12 batches	Steam: 720 kg
Seasoning	Mixing with seasonings	Double-spiral seasoning mixer, processing capacity ≥ 417 kg/h	3.55 kW × 8 h, 1 unit	Electricity: 28.4 kWh
Packaging	Vacuum packaging	Fully automatic vacuum packaging machine, capacity: 1200 packs/h per unit, 200 g/pack, 2 units	6 kW/unit × 2 units × 8 h	Electricity: 96 kWh
Sterilization	120 °C, total duration 40 min (holding for 20 min)	High-pressure sterilization retort, capacity: 600–1000 kg/batch, 556 kg/batch, 6 batches in total	Electricity: 13.5 kW × (0.67 h/batch × 6 batches); Steam: 280 kg/batch × 6 batches	Electricity: 54 kWh; Steam: 1680 kg

Note: All energy consumption values were estimated based on a daily production output of 3.334 t and the equipment operating parameters listed above. * The braising step was included in the conventional process but omitted in the DSPG-I process.

## Data Availability

The original contributions presented in this study are included in the article. Further inquiries can be directed to the corresponding author.
